# Prosodic cues enhance rule learning by changing speech segmentation mechanisms

**DOI:** 10.3389/fpsyg.2015.01478

**Published:** 2015-09-30

**Authors:** Ruth de Diego-Balaguer, Antoni Rodríguez-Fornells, Anne-Catherine Bachoud-Lévi

**Affiliations:** ^1^Institució Catalana de Recerca i Estudis AvançatsBarcelona, Spain; ^2^Cognition and Brain Plasticity Unit, Hospitalet de Llobregat, Bellvitge Research Biomedical Institute (IDIBELL)Barcelona, Spain; ^3^Department of Basic Psychology, University of BarcelonaBarcelona, Spain; ^4^INSERM U955, Equipe 01, Neuropsychologie Interventionnelle, Institut Mondor de Recherche BiomédicaleCréteil, France; ^5^Département d’Etudes Cognitives, École Normale SupérieureParis, France; ^6^Faculté de Médecine, Université Paris-EstCréteil, France; ^7^Assistance Publique-Hôpitaux de Paris, Centre de Référence Maladie de Huntington, Unité de Neurologie Cognitive, Hôpital Henri Mondor-Albert ChenevierCréteil, France

**Keywords:** prosody, segmentation, rule-learning, event-related potentials (ERPs), N1, P2, artificial language learning, statistical learning

## Abstract

Prosody has been claimed to have a critical role in the acquisition of grammatical information from speech. The exact mechanisms by which prosodic cues enhance learning are fully unknown. Rules from language often require the extraction of non-adjacent dependencies (e.g., he plays, he sings, he speaks). It has been proposed that pauses enhance learning because they allow computing non-adjacent relations helping word segmentation by removing the need to compute adjacent computations. So far only indirect evidence from behavioral and electrophysiological measures comparing learning effects *after* exposure to speech with and without pauses support this claim. By recording event-related potentials *during* the acquisition process of artificial languages with and without pauses between words with embedded non-adjacent rules we provide direct evidence on how the presence of pauses modifies the way speech is processed during learning to enhance segmentation and rule generalization. The electrophysiological results indicate that pauses as short as 25 ms attenuated the N1 component irrespective of whether learning was possible or not. In addition, a P2 enhancement was present only when learning of non-adjacent dependencies was possible. The overall results support the claim that the simple presence of subtle pauses changed the segmentation mechanism used reflected in an exogenously driven N1 component attenuation and improving segmentation at the behavioral level. This effect can be dissociated from the endogenous P2 enhancement that is observed irrespective of the presence of pauses whenever non-adjacent dependencies are learned.

## Introduction

All human languages are characterized by the presence of prosody. The hallmarks of prosody, such as vowel length, pauses, and loudness mark different rhythmic patterns in different languages. Already at an early age, infants are very sensitive to these characteristics of their mother tongue (Mehler et al., 1988; Friederici et al., 2007). This sensitivity facilitates the segmentation of words from fluent speech and the acquisition of the syntactic relations between these words (Trainor et al., 2000; Christophe et al., 2008; Shukla et al., 2011). However, the exact effects those prosodic cues have in the way we treat the speech signal to improve learning mechanisms remains unknown.

Among the different cues attributed to prosody, pauses play a particularly relevant role in language learning. The presence of pauses in speech has been shown to trigger the ability to generalize the embedded rules in artificial languages (Peña et al., 2002; Endress and Hauser, 2010; Mueller et al., 2010). However, prosody is not the only cue enhancing language learning, distributional information is crucial. Statistical learning can be used by both adults and infants to segment words based on the use of adjacent transitional probabilities between syllables (Saffran et al., 1996), and to learn non-adjacent relations (Gómez and Gomez, 2002; Newport and Aslin, 2004). Peña et al. (2002) proposed that the presence of pauses changes the way learners process the speech signal by discharging the system from processing distributional information. From this perspective, the presence of pauses may help the system to locate word boundaries more easily with no need to attend to adjacent statistical information. Once words are segmented, attention can be redirected to internal relations within words or between words laying the grounds for the creation of higher order categories indirectly enhancing rule generalization. This conclusion nevertheless has never been sustained by direct evidence so far. Only indirect evidence based on the effects of pauses on the final output of the learning process is available. This evidence indicates that subtle pauses as short as 25 ms rapidly lead to rule generalization, whereas without pauses, even much prolonged exposure to the same artificial language fails to produce rule learning. For example, Mueller et al. (2010) showed that for the acquisition of center embedded structures, which rely also on the extraction of non-adjacent relations, mere distributional information is not enough for successful learning either. Prosodic cues marking the boundaries of the major units are necessary. In addition, in another study (Mueller et al., 2008) the electrophysiological responses in correct sentences containing non-adjacent dependencies between words and rule violations were different after exposure to language with pauses marking the boundaries between sentences versus continuous presentations (despite an absence of learning differences between the two conditions). Therefore, pauses may cause a perceptual change that allows shifting learning from adjacent to non-adjacent information (Aslin and Newport, 2012).

In the current investigation we were interested in understanding how the presence of pauses changes the way speakers treat the speech signal during learning. In particular, if the presence of pauses creates a trade off between segmentation and rule learning then (i) we should be able to dissociate those electrophysiological modulations purely driven by the pauses segmenting speech exogenously irrespective of possible learning from (ii) modulations associated to the learning process irrespective of the presence of pauses. Studying the brain electrophysiological changes associated to the appearance of pauses in the speech stream *during* learning may give us critical information to understand this issue. In the current investigation we compared conditions with random presentation of syllables, where no learning could be reached, with and without prosodic information (given by rhythmic pauses between words), and conditions where words and rules could be extracted with and without pauses in order to study the effects of prosodic cues in rule learning from speech.

Based on previous work we know that online segmentation of words from an artificial continuous speech stream made of nonsense words with no embedded rules induces a N1 modulation (Sanders et al., 2002) and a progressive time-related increase-decrease N400 modulation (Cunillera et al., 2009; François et al., 2014). Because this latter component appeared to be modulated both in continuous speech and in languages with pauses (De Diego Balaguer et al., 2007), we expected to see differences specifically in N1 amplitude modulation between continuous languages and languages with pauses. Languages with pauses with embedded rules induce also a progressive modulation of the P2 component (De Diego Balaguer et al., 2007). If this component is fully determined by the simple presence of pauses then this modulation should also be present for random stream with pauses irrespective of the fact that learning cannot be accomplished in this condition. In contrast, if this component is purely driven by rule learning we should therefore observe a P2 modulation in those participants that learn rules even when prosody is not present indicating an endogenous origin for this modulation independent from the segmentation effect.

## Materials and Methods

### Participants

Twenty-three right-handed volunteers (12 men, mean age 28.5 ± 5 SD), with no history of neurological or hearing deficits, participated in the event-related potentials (ERP) experiment. Two participants were discarded from the ERP analysis due to excessive eye-movements. The participants were native Spanish speakers. The protocol was approved by the ethics committee of the University of Barcelona, and written consent from all participants was obtained prior to the experiment. Participants were paid for their participation.

### Material

Six artificial languages were used, and each language was modified to create versions for the different conditions. Four of these languages were the same speech streams previously used in De Diego Balaguer et al. (2007). The words in all of the artificial languages were trisyllabic and built such that the initial syllable in each word determined the final syllable, irrespective of the middle syllable. This structure was used to form a non-adjacent dependency similar to the morphological rules of real languages (e.g., he plays, he sings, he speaks; in the artificial language: e.g., **ba**pi**gu**, **ba**fe**gu**, **ba**lo**gu**). There were three different rule frames (**ba**__**gu**, **do**__**ke**, and **mo**__**ti**) and three different syllables (i.e., fe, pi, lo) could intervene as middle elements combined with all three frames, leading to nine different words for each structured language, consistent with the same principles described in Peña et al. (2002). To avoid interference during learning, none of the syllables were repeated across languages.

The streams and test items were synthesized using the MBROLA speech synthesizer software (Dutoit et al., 1996), concatenating diphones at 16 kHz from the Spanish male database (es2)^1^. The words lasted 696 ms each and were separated by 25-ms pauses (Peña et al., 2002; De Diego Balaguer et al., 2007). Therefore, pauses were consistently inserted at positions where syllables carrying the rule dependency appeared, although the participants were not aware of their presence, as informally reported after the experimental session. No other prosodic information was inserted. The words were concatenated in pseudo-random order to avoid immediate repetition of the same structure. Therefore, transitional probability between the first and the last syllable of every word was 1.0, while the corresponding probability between the last syllable of any word and the first syllable of the following word was 0.5. As the same middle syllables appeared in the three frames of a given language, the transitional probability between the initial and middle syllable, or between the middle and the final syllable was 0.33.

**FIGURE 1:**
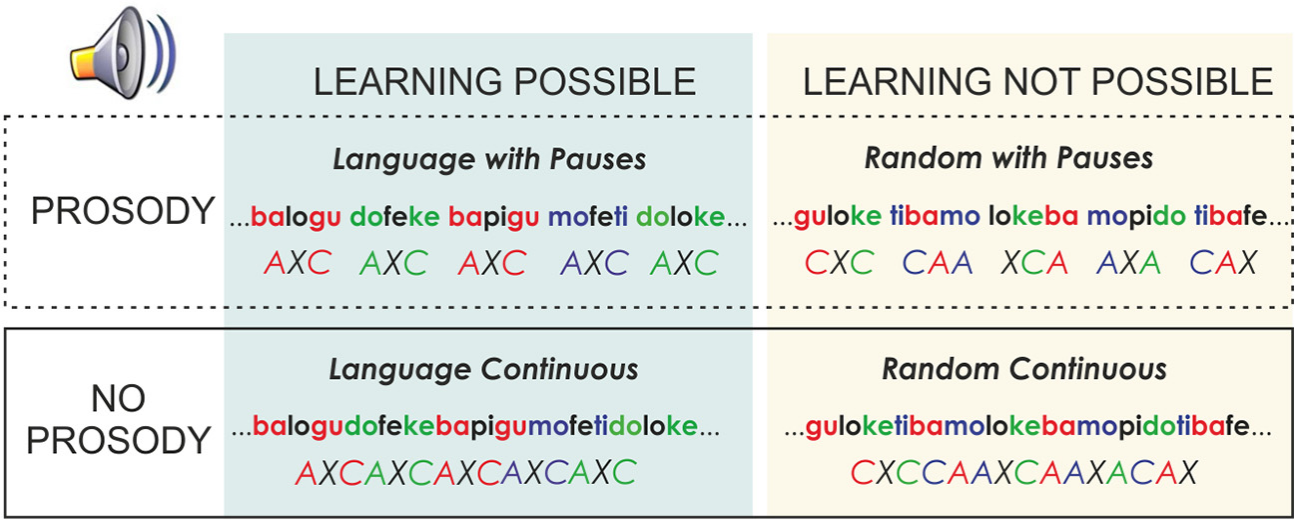
**Scheme of the different experimental conditions used, where the crossed factors (Learning and Prosody) are illustrated**.

Each language of the material with pauses was modified to create a version without pauses. In addition, each of these versions had a random counterpart (**Figure 1**). In this random version, the same syllables of each language were concatenated in pseudo-random order, with the constraint that a syllable was never immediately repeated. Therefore, the transitional probability between all syllables in the Random conditions was 0.12. Thus, under these conditions, no words or rules could be learned as no prediction of the following syllable can be derived. In addition, as in the Language version, half of the Random streams also included 25-ms pauses every three syllables and the other half were presented continuously. This manipulation allowed observing the effects of pause insertions on the speech stream, independently of learning. Under all conditions, the words appeared the same number of times in languages with and without pauses.

### Procedure

The experiment involved *learning* and *test* phases. Each participant heard a total of four structured languages and two random streams; half of the languages were presented with pauses and the other half were presented without pauses (**Figure 1**). The order of presentation was counterbalanced across subjects with half of the orders initiated using a language with pauses and the other half initiated with a continuous language. The same subject never heard more than one version of a language using the same syllables. During the *learning phase* of the experiment, each speech stream was presented for 4 min leading to 336 trisyllabic observations per speech stream. This led to 336 total observations for random with pauses, 336 for the random continuous condition, 672 for the language condition with pauses and 672 for the language condition continuous. Participants were informed that they would hear a nonsense language and they would be subsequently asked to identify the words of this language.

Immediately after each language exposure, the participants were behaviourally tested using a two-alternative forced choice test (*test phase)* with isolated test items presented in pairs. One stream from each condition was tested for *word acquisition*, such that in each trial, the participants had to choose between words from the exposed language and non-words. Non-words were new items formed using the same three syllables of a previously exposed word in an incorrect order (e.g., **gu**fe**ba**). The other stream from each condition was tested for *rule learning*, such that participants had to choose between a non-word and a rule-word. Rule-words were new words with the same initial and final syllables, therefore following the same structure as used in the exposed language, with a syllable corresponding to another word inserted in the middle position (e.g., **ba**do**gu**). Thus, both non-words and rule-words were new words created using the same syllables and violating the transitional probability on adjacent syllables; however, only rule-words followed the structure of words in the artificial languages. Each test item (9 words, 9 rule-words, and 18 non-words) appeared three times in each recognition phase. In the case of random streams, there are no rules or words; thus, half of the participants received the test with words after the continuous random and with rules after the random with pauses condition and the other half of the participants received the reverse. All experiments were run on a PC computer, using Presentation software^2^, and the sounds were presented through headphones.

### EEG Acquisition

EEG was acquired during the learning phase. The ERPs were recorded from the scalp at 29 standard locations (Fp1/2, Fz, F7/8, F3/4, Fc1/2 Fc5/6, Cz, C3/4, T3/4, Cp1/2, Cp5/6, Pz, P3/4, T5/6, Po1/2, and O1/2) with a BrainAmp system (Brain Products GmbH). In addition, the vertical and horizontal eye-movements were monitored, respectively, using an electrode at the infraorbital ridge of the right eye and at the outer canthi of the eyes. The electrode impedances were maintained below 3 kOhm. The electrophysiological signals were filtered with a bandpass of 0.01–50 Hz (half-amplitude cut-offs) and digitalised at a rate of 250 Hz. The biosignals were referenced online to the electrode in the outer canthus of the right eye and re-referenced off-line to the mean of the activity at the two mastoids. Trials with base-to-peak electro-oculogram amplitude of more than 50 μV, amplifier saturation, or a baseline shift exceeding 200 μV/s were automatically rejected off-line. Stimulus-locked ERPs were averaged for epochs of 1024 ms initiated at 100 ms prior to the stimulus. The mean rejection rate in the final sample of participants analyzed was 24.6 trials ± 16.2.

### Data Analysis

For the EEG analyses, the ERP responses for all word presentations that appeared during the 4 min of exposure, from stimulus onset, were pooled together in each language and then averaged across the different languages used for each condition. The same average in the random conditions corresponded to all trisyllabic presentations bounded between pauses pooled together in the condition with pauses and the corresponding trisyllabic presentations for the continuous condition.

For both the ERP we used a baseline from -50 ms to 0 (stimulus onset) and we considered two within subjects factors in the ANOVA: Prosody (Continuous vs. with Pauses) and Learning (Language vs. Random) associated with the type of material presented in the learning phase and then specific pair-wise comparisons within each condition were performed to test each of the specific hypothesis. For the behavioral data an ANOVA with Prosody and Type of Test (Words vs. Rules), associated with the nature of the information tested, was performed (see Materials and Methods; **Figure 1**). Details of the additional analyses are described in detail in the Results section. In the EEG analysis, each ANOVA was performed for the critical time-windows at parasagittal (PS) [five levels for the anterior–posterior factor (AP: Fp1/Fp2, F3/F4, C3/C4, P3/P4, O1/O2)] and temporal (TE) locations [three levels for the anterior–posterior factor (AP: F7/F8, T3/T4, T5/T6)], including the hemisphere factor (Hem: left, right), and midline (ML) locations [three levels for the anterior–posterior factor (AP: Fz, Cz, and Pz)]. The Huynh-Feldt epsilon correction was used when appropriate, and the corrected *p*-value is reported. Based on visual inspection, the analyses for the N1 component amplitude was performed at the 90–140 ms post-stimulus onset, where the peak of the component was observed in the present study. This time-window was within the range previously used in similar studies of this component (Sanders et al., 2002; Astheimer and Sanders, 2009). The 140–190 ms time-window was assessed to evaluate the effect of the P2 amplitude. In specific comparisons where the peak of the differences observed is delayed from this time window, the window was moved to fit the peak latency. The time-window was nevertheless always moved within the range of the 120–230 ms in agreement with previous studies (Cunillera et al., 2006; De Diego Balaguer et al., 2007) and then specified in the results section. The auditory vertex potential that contains the N1 and P2 is triggered by sound onset and therefore there is debate in the auditory literature concerning whether these components can be labeled as N1 and P2 in continuous speech (Lalor et al., 2009; Lalor and Foxe, 2010). We keep the same nomenclature for continuous streams and streams with pauses for the sake of comparison between them and with the previous literature on the topic that did use these labels for the description of the effects (Sanders et al., 2002; Abla et al., 2008; Cunillera et al., 2008, 2009; Astheimer and Sanders, 2011).

In addition, in the comparisons between conditions with pauses to conditions continuous, in order to ensure that the effects were not due to modulations previous to baseline we ran further analyses with a longer baseline (-100 to 0 ms) on a region of interest (ROI) where the N1 and P2 effect were maximal (i.e., F3, F4, C3, C4). We ran stepwise two-tailed serial *t*-tests (step size = 4 ms) on the comparison between each condition continuous and with pauses following Rodriguez-Fornells et al. (2002) procedure. For each test, data were averaged in 40 ms windows and tested every 4 ms (i.e., *t*-test were conducted on averages from 0 to 40 ms, 4 to 44 ms, 8 to 48 ms, etc.), from -100 to 604 ms from the onset of the trisyllabic presentation. A correction was then applied so that only clusters of at least four consecutive *t*-tests at (*P* < 0.05) were considered significant. The onset of the effect was determined as the onset of the *t*-test time window at which the four following consecutive tests were significant in this cluster.

Since baseline differences were present in these analyses we carried out additional analysis on the peak-to-peak difference for the components of interest to obtain amplitude measures independent of baseline activity (Luck, 2004). Peak to peak amplitudes were extracted in the ROI for each subject for the N1 components of each syllable in the random conditions and for the N1 and P2 components of the first syllable for the language conditions. For the N1 component the amplitude was calculated for each subject as the difference between the maximal positive value in a time-window of 50 ms around the most positive peak previous to the N1 and the most negative peak in a 50 ms window around the peak of the N1. For the P2, since the N1 component was greatly attenuated in both language conditions and actually not detectable in the language condition with pauses, we could not use the N1 amplitude for the calculation of the P2 peak-to-peak amplitude (Luck, 2004). We therefore calculated the amplitude as the difference between the maximum negative peak in a 50 ms window around the peak of the first clear negative deflection in the epoch to the maximum positive amplitude in the 50 ms window around the peak of the P2 component. Pairwise comparisons were then performed between the amplitudes in the conditions continuous and with pauses.

## Results

### Learning Performance

**FIGURE 2:**
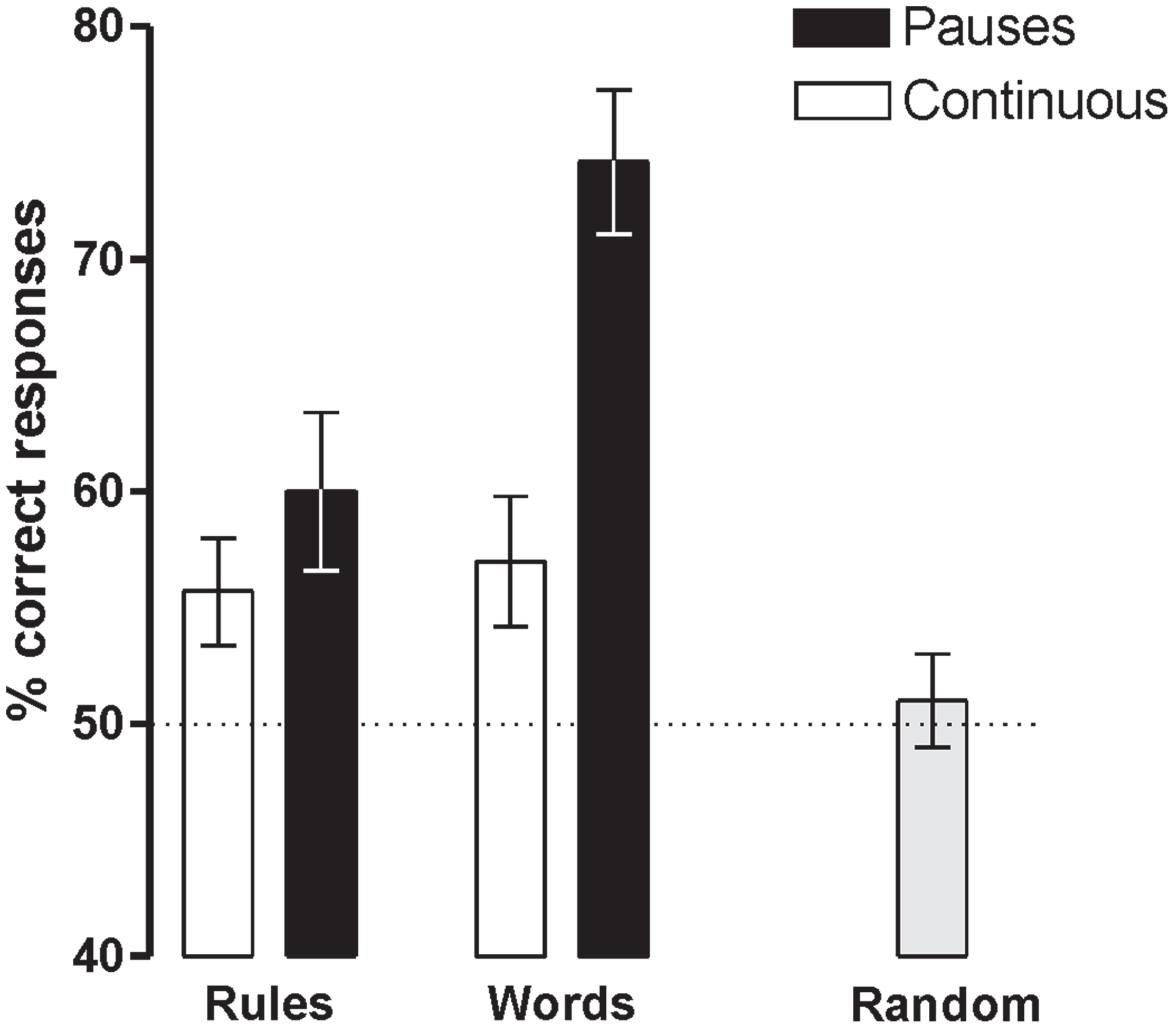
**Word and rule-learning performance measured after the 4 min of language exposure (with pauses, continuous, and random)**.

Participants were able to learn words and rules above chance (50%) in both Prosody conditions [Language with Pauses: Words 73.7% ± 3.2, *t*(18) = 7.31, *P* < 0.0001, *d* = 1.67 and Rules 58.5 ± 3.3, *t*(18) = 2.59, *P* < 0.018, *d* = 0.59; Language Continuous: Words 56.6 ± 2.9, *t*(18) = 2.24, *P* < .038, *d* = 0.51 and Rules 55.3 ± 2.4, *t*(18) = 2.19, *P* < 0.042, *d* = 0.50] (**Figure 2**). However, the presence of pauses enhanced word learning more than rule generalization, as indicated by the interaction between Prosody and Type of Test [*F*(1,17) = 10.04, *P* < 0.006, $\eta_{p}^{2}$ = 0.36]. While rule learning did not differ in the two Prosody conditions (*P* > 0.3, *d* = 0.25), word learning was greater in languages with pauses than in continuous languages [*t*(18) = -5.65, *P* < 0.0001, *d* = 1.08]. Learning was at chance levels in Random conditions (mean 52.1% ± 8.9; *P* < 0.3, *d* = 0.24).

### ERP Results

**FIGURE 3:**
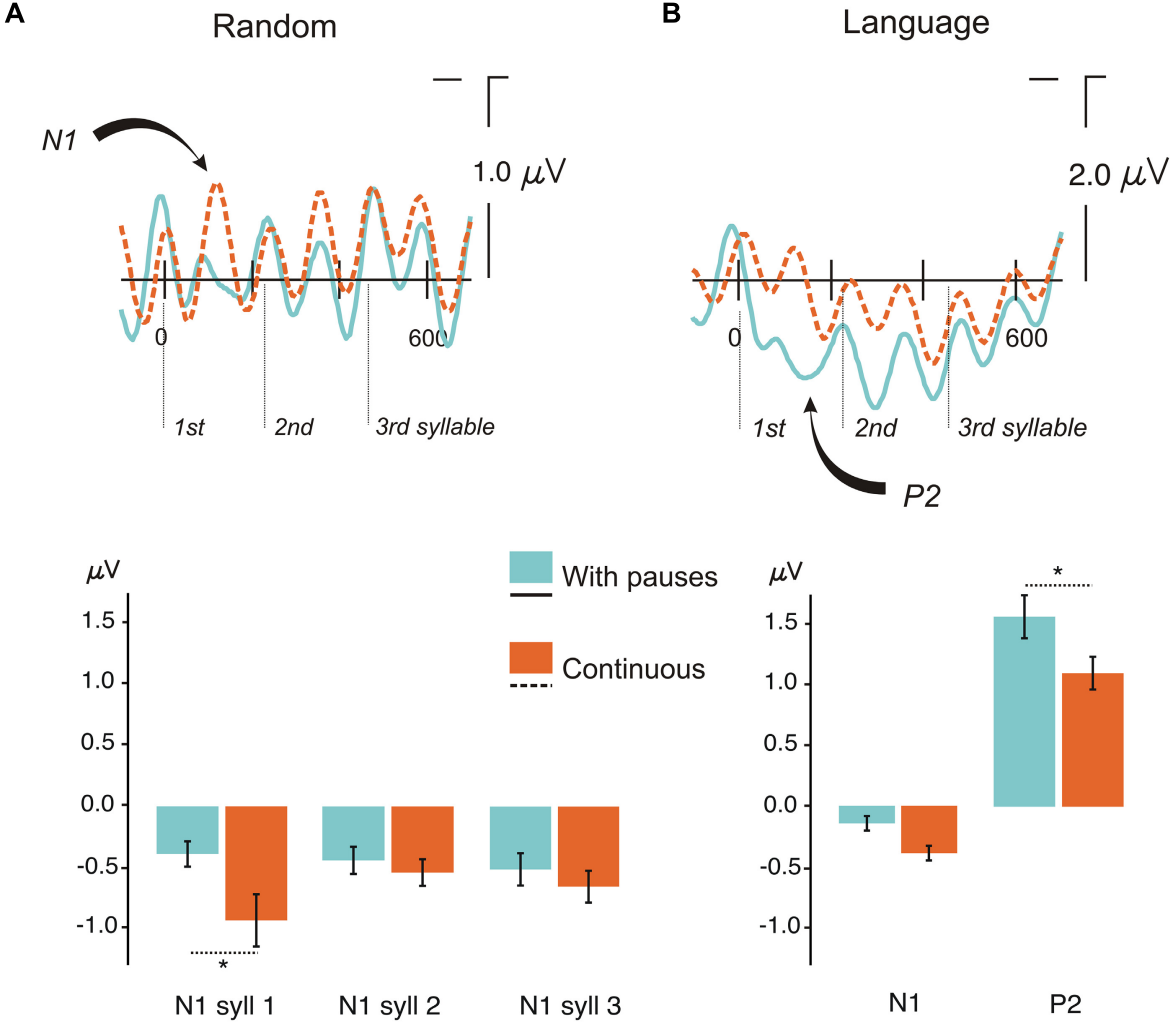
**Event-related potentials (ERP) results for the conditions continuous and with pauses. (A)** Top: Grand Average ERPs for each trisyllabic presentation for random streams in the continuous condition and the condition with pauses at the ROI (average of F3, F4, C3, C4 electrodes) analyzed in the serial *t*-test analysis with a baseline from -100 ms to stimulus onset. Bottom: Mean peak-to-peak differences from the previous positive peak to the N1 peak amplitude for each syllable in the random condition with pauses and continuous. Bars indicate standard error of the mean (SEM). **(B)** Top: Grand Average ERPs for each trisyllabic word for language streams in the continuous condition and the condition with pauses at the ROI (average of F3, F4, C3, C4 electrodes) analyzed in the serial *t*-test analysis with a baseline from -100 ms to stimulus onset. Bottom: Mean peak-to-peak differences from the previous positive peak to the N1 peak amplitude and from the first negative peak in the epoch to the peak amplitude of the P2 component in the language condition with pauses and continuous. Bars indicate SEM. ^∗^*P* < 0.05.

The global ANOVA performed with Prosody (Continuous and with Pauses) and Learning (Language and Random) as within-subject factors is summarized in **Table 1**. Overall, Language streams differed from their Random counterparts with a sustained positive going wave observed in Language streams when compared to Randoms (see **Figure 3**; comparison between the dotted lines in panels A vs. B and between the solid lines). Interestingly, Language vs. Random comparisons showed earlier differences in the streams with Pauses than in Continuous streams (see **Table 1**, Learning effect × Prosody significant interactions for the N1 time-window). Indeed, a finer-grained moving window analysis in steps of 10 and 50-ms epochs from a 10–60 ms post-stimulus onset, indicated that Language streams with pauses started to differ from their Random counterparts from the earliest time-window [ML: *F*(1,20) = 25.07, *P* < 0.0001; PS: *F*(1,20) = 21.06, *P* < 0.0002, Learning × AP: *F*(4,80) = 3.56, *P* < 0.03; TE: *F*(1,20) = 11.83, *P* < 0.003, Learning × AP: *F*(2,40) = 4.13, *P* < 0.03]. The AP interaction at PS sites reflected that this effect peaked at F3/F4 [*F*(1,20) = 20.86, *P* < 0.0002] and was significant at all locations (all *P*s < 0.025) except for the most posterior electrodes O1/O2 (*F* < 1). At TE sites, the interaction reflected also a significant effect at all electrodes (all *P*s < 0.015) except for T5/T6 (*F* < 1) and peaking at F7/F8 [*F*(1,20) = 16.12, *P* < 0.0007].

**Table 1:** Summary of the overall ANOVA for the mean amplitudes in the random and language conditions, comparing the conditions with pauses to continuous conditions at different time windows.

	90–140 ms			140–190 ms		
	ML	PS	TE	ML	PS	TE
Learning^a^	26.77^+++^	24.95^+++^	23.90^+++^	54.77^+++^	34.70^+++^	18.89^+++^
Prosody^a^	40.00^+++^	35.50^+++^	24.67^+++^	16.64^+++^	15.39^+++^	10.83^+++^
L × P^a^	9.03^++^	6.90^+^	5.73^+^			
L × AP^b^	16.78^+++^	15.48^+++^	8.72^++^	24.15^+++^	20.88^+++^	12.21^+++^
P × AP^b^	11.63^+++^	18.18^+++^	16.34^+++^		6.87^+++^	4.56^+^
L × P × AP^b^	5.10^+^	3.76^+^	5.01^+^			
**Random streams**						
Prosody^a^	11.65^+++^	10.09^+++^	4.82^+^	6.48^+^	4.46^+^	
P × AP^b^		4.97^++^	4.26^+^			
**Language streams**						
Prosody^a^	45.49^+++^	38.88^+++^	31.31^+++^	11.24^+++^	10.92^+++^	12.81^+++^
P × AP^b^	16.67^+++^	21.29^+++^	22.91^+++^	7.24^++^	7.70^+++^	6.24^+++^
P × H^a^	——		6.19^+^	——		

*The numbers in the table correspond to *F-*values. Only significant values of *P* < 0.05 (Huynh-Feldt corrected) are included. Degrees of freedom for the *F* values: ^*a*^1, 20; ^*b*^2, 40 for ML and TE; ^*b*^4, 80 for PS; ^+^*P* < 0.05, ^++^*P* < 0.01, ^+++^*P* < 0.005. “—”: not applicable. L, Learning factor (Language vs. Random streams); P, Prosody factor (with Pauses and Continuous); H, Hemisphere; AP, Anterior–Posterior*.

In contrast, Continuous Language streams started to differ from their Random counterparts at a later time, from the 100–150 ms time window [ML: Learning x AP: *F*(2,40) = 3.46, *P* < 0.04; 110–160 ms: PS: Learning × AP: *F*(4,80) = 3.84, *P* < 0.03; 140–190 ms: ML: *F*(1,20) = 6.94, *P* < 0.02, Learning × AP: *F*(2,40) = 3.54, *P* < 0.04; PS: *F*(1,20) = 5.78, *P* < 0.02, Learning × AP: *F*(4,80) = 5.29, *P* < 0.003] extending to TE sites in the 320–370 ms time window [*F*(1,20) = 5.32, *P* < 0.03]. The AP interactions showed a shift in the topography of the effects in the different time windows. In the 100–150 ms time window, effects were located at the posterior and central sites of ML electrodes [Cz: *F*(1,20) = 11.57, *P* < 0.003, Pz: *F*(1,20) = 6.43, *P* < 0.02] and non-significant at Pz (*P* < 0.2). In the following time window at 110–160 ms, the effects were focused only at the most frontal sites for PS electrodes [Fp1/Fp2: *F*(1,20) = 4.58, *P* < 0.045; all other *P*s > 0.07]. At the 140–190 time-window the AP interactions reflected that the effect was widespread in all PS (all *P*s < 0.049) and ML locations (*P*s < 0.024), peaking at F3/F4 [*F*(1,20) = 7.44, *P* < .019] and Fz [*F*(1,20) = 8.45, *P* < 0.009] but were not significant at the most posterior sites [O1/O2: *F* < 1; Pz: *F*(1,20) = 4.29, *P* < 0.052].

#### Effect of Prosody in Non-Learning Conditions (Random Conditions)

The crucial comparison of the two Random conditions (Random with Pauses vs. Random Continuous) (**Table 1**, **Figure 3A**) revealed the immediate effect of presenting a small pause in the auditory stream when no learning could be accomplished, thus evaluating only the exogenous effects elicited by the presence of pauses. As shown in **Figure 3A** (top), the presence of pauses clearly reduced the mean amplitude of the N1 component in the first syllable compared with the Continuous condition (**Table 1** for statistical analyses) (see **Figure 3A**, top). The topographical distribution of this auditory N1 modulation showed a standard fronto-central distribution reflected by the Prosody × AP interaction (**Table 1**). The effect was significant at all PS and TE sites (all *P*s < 0.026) except at posterior sites (O1/O2 and T5/T6, *F* < 1), peaking at F3/F4 [*F*(1,20) = 12.83, *P* < 0.002] and C3/C4 electrodes [*F*(1,20) = 15.57, *P* < 0.0008].

**FIGURE 4:**
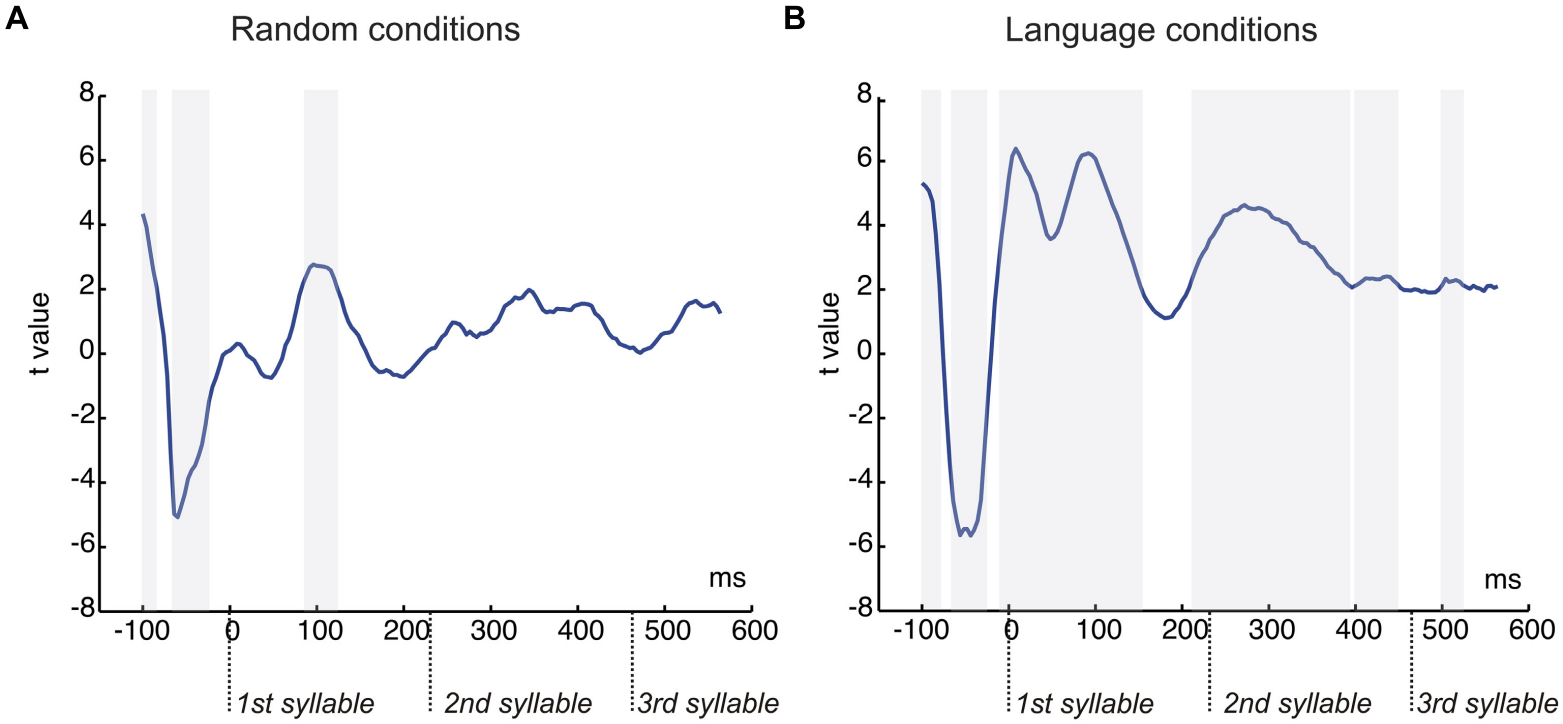
**Graphical representation of the serial *t*-test analysis in 4 ms steps comparing the conditions with pauses and continuous. (A)** *T*-tests values for the comparison of random streams continuous and with pauses. **(B)** *T*-tests values for the comparison of language streams continuous and with pauses. Each time point corresponds to a time-window of 40 ms. Gray shaded areas correspond to significant points (*P* < 0.05) in clusters of at least four consecutive points, shaded from the onset of the cluster.

The results of the stepwise one-tailed serial *t*-tests in the ROI with a -100 to 0 ms baseline, on the comparison between the random streams continuous and with pauses showed that the onset of the N1 effect arose at 84 ms for the first syllable lasting until 120 ms. The differences in this component in the second and third syllables were not significant (**Figure 4A**). This analysis showed also that clear significant differences were present between the conditions with pauses and continuous (from -100 to -88 and from -68 to -28 ms, **Figure 4A**). Therefore a peak-to-peak analysis was performed to control for the influence of this effect on the N1. The amplitude differences remained significant for the first syllable [*t*(20) = 2.13, *P* < 0.046] and were not observed in the second and third syllables (both *P* > 0.1) in this analysis (**Figure 3A**, bottom).

#### Effect of Prosody in Learning Conditions (Language Conditions)

As for the Random conditions, the comparison between the two Language conditions showed a decrease in the amplitude of the N1 component in conditions with Pauses (**Figure 3B**, top; **Table 1** for statistical analyses). This N1 difference was only observed at the first syllable (**Table 1**) corresponding to the word onset. As in the case of random streams the N1 effect showed a frontocentral distribution reflected in a Prosody × AP interaction (**Table 1**). The effect was significant at all ML, PS, and TE electrodes (all *P*s < 0.00001) but more reduced at posterior sites [O1/O2: *F*(1,20) = 7.94, *P* < 0.011; T5/T6: *F*(1,20) = 5.82, *P* < 0.026].

Importantly, a second modulation, which was not present in the Random streams, was observed. Here, the N1 attenuation was followed by an increase in the amplitude of the P2 component (see **Figure 3B**, top; **Table 1**) in Languages with Pauses, with a frontal topographic distribution reflected in a Prosody × AP interaction (**Table 1**). The P2 effect was significant at all ML, Ps, and TE electrodes (all *P*s < 0.006), more reduced at Fp1/Fp2 [*F*(1,20) = 4.92, *P* < 0.038] and non-significant at posterior sites (O1/O2 and T5/T6, *P*s > 0.07).

The stepwise one-tailed serial *t*-tests analysis, with the longer -100 to 0 ms baseline, showed that the difference between conditions arose since the baseline period (see **Figure 3B**, top and **Figure 4B**), overlapping with the N1 and P2 effects. The differences were significant until 192 ms after stimulus onset. Additional differences were observed after 212 to 393 ms and 440 to 448 ms (spanning the end of the first syllable and the second), and 500 to 524 ms (in the third syllable) (**Figure 4B**). Given the effects in the baseline and the spill over effects in the time windows of the N1 and P2 components, here again a peak-to-peak approach was used to control of these effects. The N1 amplitude difference between the continuous condition and the condition with pauses did not reach significance [*t*(20) = 1.63, *P* > 0.1] (**Figure 3B**, bottom) probably due to the important attenuation of the N1 component in both continuous and random conditions compared to N1 amplitudes previously observed in the random counterparts [Random vs. Language Continuous: *t*(20) = 1.99, *P* < 0.060; Random vs. Language with Pauses: *t*(20) = 2.66, *P* < 0.015]. Nevertheless, it is worth noting that the N1 amplitude was not different from 0 in the condition with pauses [*t*(20) = 1.39, *P* > 0.1] whereas the N1 component was clearly present in the continuous condition [*t*(20) = 4.48, *P* < 0.0001]. The P2 peak-to-peak difference between language conditions continuous and with pauses was significant in the peak-to-peak analysis [*t*(20) = 2.27, *P* < 0.034] (**Figure 3B**, bottom).

**FIGURE 5:**
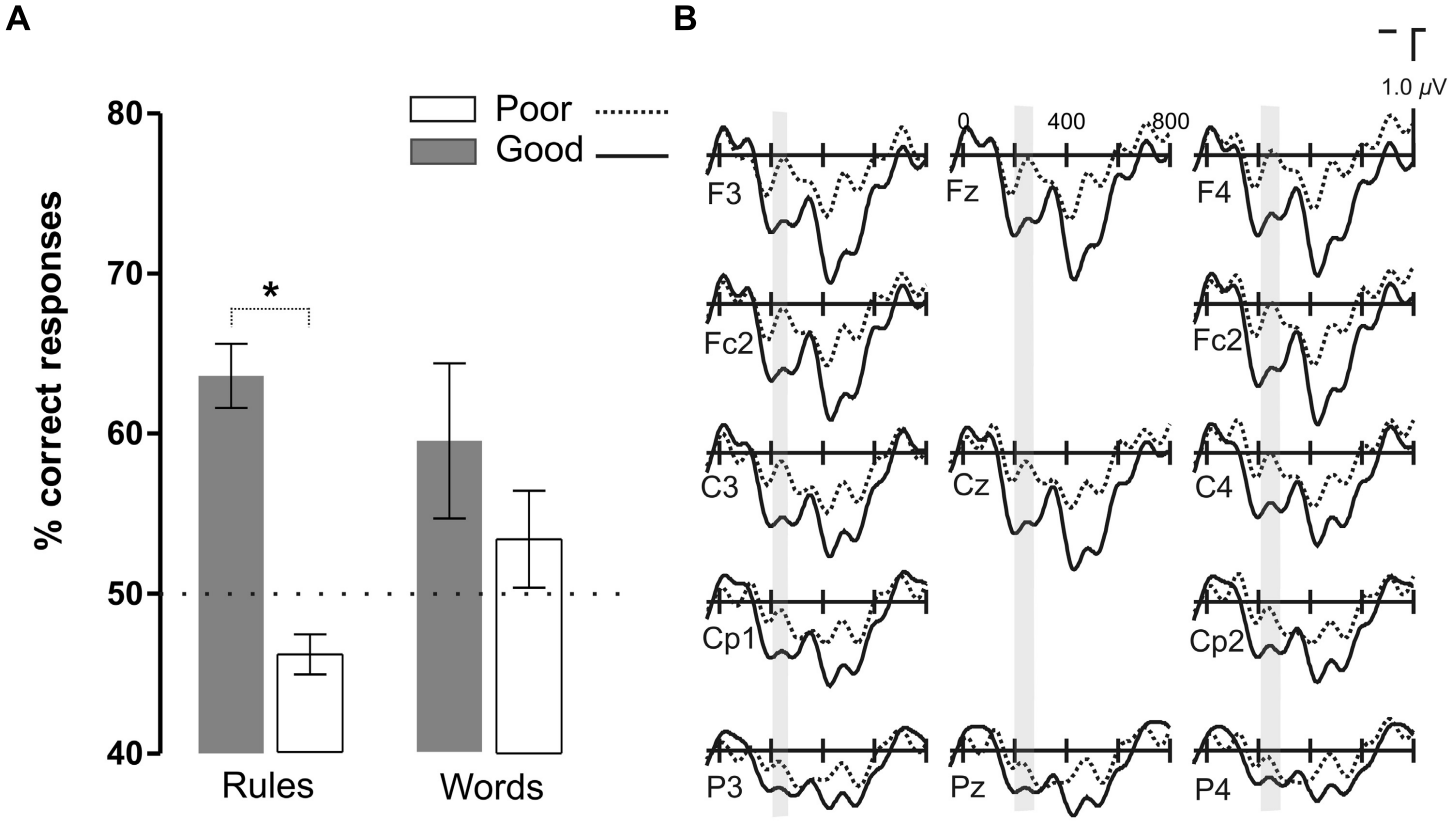
**Results of the comparison between the two groups of participants split as a function of their rule learning performance in Continuous Language condition. (A)** Word and rule-learning performance in the two groups of participants. The groups differed in terms of their rule learning performance but were matched in their word learning performance. **(B)** Grand average ERP waveforms corresponding to good (Good) and bad (Poor) rule learners in the Continuous Language condition.

In order to test whether this P2 modulation was specific to the presence of pauses or whether it corresponded to a more endogenous component related to the learning process, we divided the participants in two groups according to their accuracy in rule learning performance but in the Continuous Language condition where no pauses could be used as exogenous cues for learning (**Figure 5**). Participants were median split (median: 55.6%) according to their performance in the rule learning test in the Continuous Language condition [mean: 63.6% ± 6.3 for Good learners, 46.1% ± 4 for Poor learners; *t*(18) = 7.4, *P* < 0.0001]. The participant with the greatest performance in word learning was removed from the Good learner group to match the two groups in word learning performance (*P* > 0.1). While electrophysiological responses did not differ in the earliest time-window corresponding to the N1 component (ML, PS and TE: *P* > 0.1), the Good learner group showed a larger P2 amplitude. The difference in the P2 appeared between 200 and 230 ms [ML: *F*(1,18) = 4.26, *P* < 0.054; PS and TE: *P* > 0.1], greatest at Cz [*F*(1,18) = 5.07, *P* < 0.037], later than in the previous comparison, corresponding to the moment where the increased positivity in the Good learner group coincides with an increased negativity in the Poor learner group (see **Figure 5**).

#### Effects of Prosody in the Course of Learning

**FIGURE 6:**
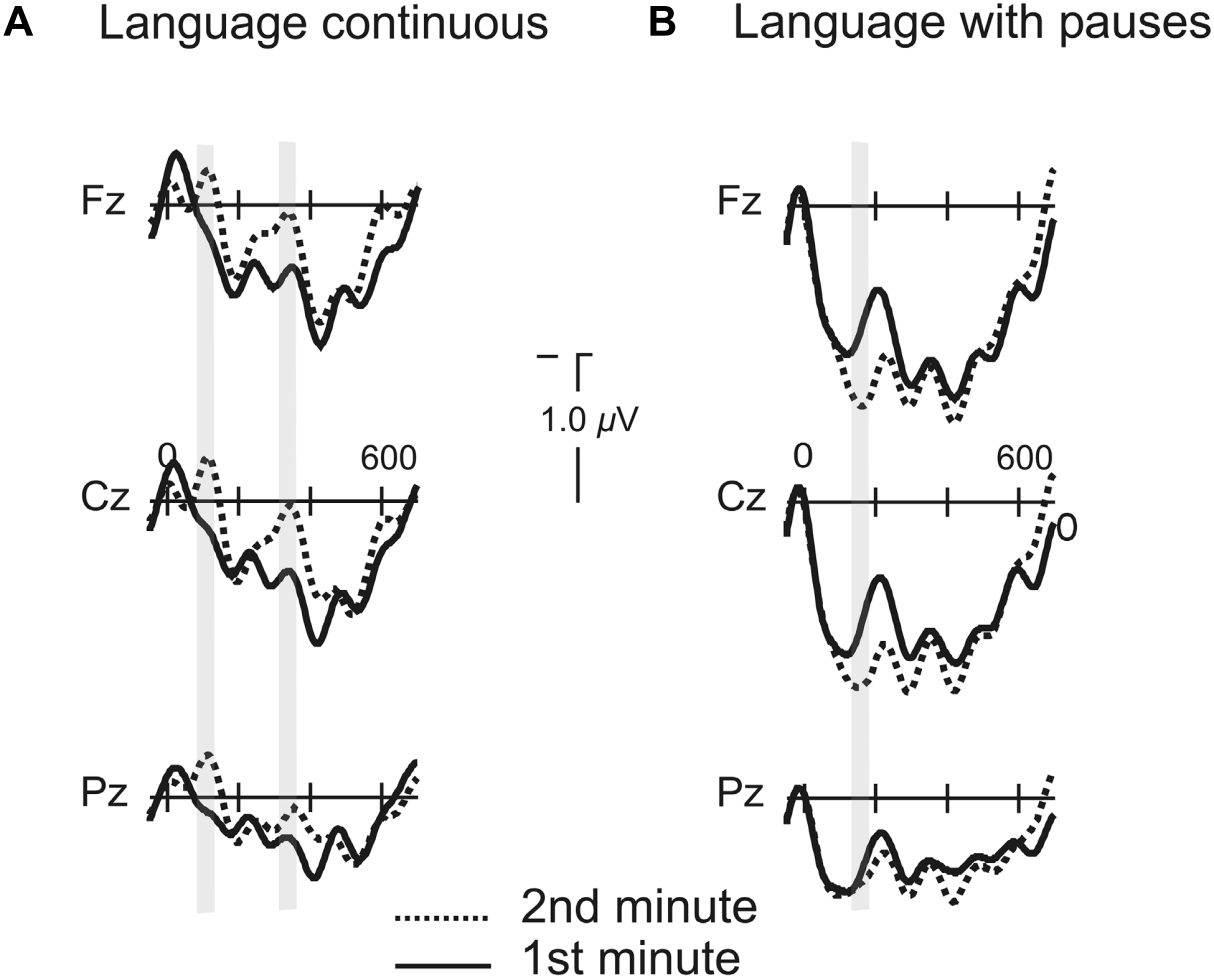
**Grand average of the ERPs for the first and second minutes of exposure to the languages continuous and with pauses. (A)** Grand average of the ERPs for the first and second minutes of exposure to the language continuous. Gray shaded marks indicate the time window of the N1 for the first and second syllables. **(B)** Grand average of the ERPs for the first and second minutes of exposure to the language with pauses. Gray shaded marks indicate the time window of the P2 component from word onset.

In order to grasp whether the progressive exposure to the language induced different modulations as a function of the presence or absence of pauses we compared the averaged ERPs for all words during the first minute of exposure to the average of all words presented during the second minute of exposure for the two conditions where learning was possible (Language with Pauses and Continuous). In the Continuous Language, a broadly distributed modulation in the N1 component appeared in the second minute of exposure [ML : *F*(1,20) = 12.08, *P* < 0.002; PS : *F*(1,20) = 11.80, *P* < 0.003; TE: *F*(1,20) = 5.95, *P* < 0.024] while no modulation was present in the time-window corresponding to the P2 (*P* > 0.1) (**Figure 6**). The modulation was present also in the time-window of the N1 from the second syllable onset [ML: *F*(1,20) = 4.63, *P* < 0.044; PS: *F*(1,20) = 3.56, *P* < 0.074; TE: *P* > 0.1]. The Language with Pauses showed also very rapid modulations (**Figure 6**). While N1 modulations were not present in this condition, a progressive P2 increase was observed, with a slightly later peak corresponding to the 170–220 ms time-window [ML: *F*(1,20) = 5.06, *P* < 0.036; PS: *F*(1,20) = 3.85, *P* < 0.064; TE: *P* > 0.1].

## Discussion

In the present study, we showed that the presence of subtle prosodic pauses induces changes in the way the brain treats the speech signal. The results indicate that the presence of pauses in speech promoted neural modulations in different ERP components for word segmentation and for rule learning.

### Effects of the Prosodic Cue on Speech Segmentation

In particular, the presence of pauses in speech induced an early effect in the ERP responses: a decrease in the amplitude of the N1. Outside the language domain, clear increases in the amplitude of the N1 are observed for selectively attended material, and reductions in amplitude are observed for expected, ignored or suppressed materials (Hillyard et al., 1973; Woldorff and Hillyard, 1991; Lange, 2010). Consistently, in the present study, the N1 component increased in continuous streams where syllable onsets needed to be attended to track the order of syllables to locate word boundaries. We could observe that with increased exposure to the continuous language a progressive N1 increase was also observed, an effect that was not present with progressive exposure to languages segmented by pauses. In similar studies using continuous streams with newly trained words (Sanders et al., 2002) or tone sequences (Abla et al., 2008), the participants with higher performances displayed greater N1 amplitudes for the first syllable/tone. This selective attention to word onsets in speech segmentation is consistent with studies indicating that listeners dynamically allocate their attention to time-windows containing relevant information from speech during language comprehension (Astheimer and Sanders, 2009).

In contrast, the N1 component was reduced for the first syllable onsets when pauses automatically parsed the speech stream irrespective of whether learning was possible or not (**Figure 3**). This effect indicates that the presence of even subtle boundaries such as the ones used in the present study discharges the system from orienting attention to each syllable onset because pauses automatically segment the speech stream. When pauses were inserted, boundaries were automatically located and the N1 component to the first syllable was reduced. This happened for both random and language streams. Although a seemingly similar phenomena seems to occur in continuous speech when learning is overcome, since a reduction of the N1 component was observed compared to its random version (**Figures 3** and **6**), importantly, the N1 component remains to be present in this condition. In contrast, the N1 component is no longer present and it is not modulated by progressive exposure in the language segmented by pauses.

Therefore N1 enhancements appear to relate to an increased attention to syllable onsets necessary for the calculation of transitional probabilities in continuous languages. When pauses are introduced segmentation can be performed based on the pauses leading to an attenuation of the N1 component for the first syllable. Indeed, the results of the progressive differences through exposure to the language clearly show how when pauses are not available greater exposure induces increased amplitude of the N1 at word onset whereas instead of this effect, the presence of pauses induces progressive increases of the P2 component.

### Effects of the Prosodic Cue Related to Rule Extraction

One of the primary goals of this study was to understand the role of pauses in the extraction of rule dependencies. As we just mentioned, the ERP data revealed increased amplitude in the P2 component in Languages with Pauses (**Figure 3A** and **6**). The P2 modulation observed in the present study is consistent with previous findings showing increased P2 amplitude as a function of rule learning (De Diego Balaguer et al., 2007), with the same fronto-central topographic distribution. However, the results of the present study provided further information on the specific mechanisms underlying this effect. These results showed that this effect was not exogenously induced through the detection of the pauses, as no P2 modulation whatsoever was observed in random streams with pauses where learning was impossible. In addition, participants who learned the rules, even in the absence of this external prosodic cue, showed greater P2 amplitude compared with those that did not learn the rules, indicating the endogenous nature of this component (**Figure 5B**). This result was not a general effect due to greater learning abilities in better learners of the rule because in contrast to this effect, the N1 component did not differ between the two groups and the two groups were matched in their segmentation performance.

The fact that the enhancement of the P2 component is specifically related to learning is consistent with several studies showing the modulation of this component when concurrent cues are predictive of upcoming information in linguistic material. As in natural language, where prosodic boundaries correspond to syntactic boundaries (Christophe et al., 2008; Seidl and Cristià, 2008), in our experiment, the pauses were correlated to the presence of the dependencies. The same P2 modulation has been observed in color boundaries corresponding to syllable boundaries in printed words (Carreiras et al., 2005), and in correct verb agreement (de Vega et al., 2010). A similar increase in the amplitude of the P2 component was also observed when the stress pattern could be used as a word segmentation cue (Cunillera et al., 2006) and in expert musicians using a melody cue for word segmentation in a continuous speech stream (Francois and Schön, 2011). Interestingly, this effect was also observed in real language when a prosodic cue predicts the application of a specific grammatical rule (Roll and Horne, 2011). Thus, the P2 modulation observed in the present study might reflect the detection of predictive information, either a specific syllable predicting the non-adjacent one when no pauses are available but learning is accomplished or the use of the associated prosodic cue predictive of the rule dependency.

Important to the present research, several studies have shown that the presence of prosodic information *per se* is not enough to induce learning. Pauses should surround the location of the segments carrying the rule dependencies to allow learning (Endress et al., 2005; Mueller et al., 2010). This association is critical, given that research on perceptual learning has shown that the presence of correlated cues enhances implicit learning (Seitz and Watanabe, 2003; Seitz and Dinse, 2007). Therefore, this correlated prosodic information might serve as a relevant cue for language acquisition. In perceptual learning paradigms, attention to an irrelevant feature associated with the relevant information enhances saliency and perceptual learning, even when the information learned is unattended and below the threshold of perception before learning (Seitz and Watanabe, 2003). In the case of speech, pauses automatically parse the speech signal highlighting the following syllable, even when they are not directly relevant for rule-learning. However, as in the case of perceptual learning, the pauses are associated with the elements to be learned. According to the Seitz and Dinse framework (Seitz and Dinse, 2007), attention controls what is learned by determining which aspects are allowed and which aspects are restricted to further processing. Attention influences learning by boosting stimulus signals to surpass the perceptual/saliency threshold. The authors propose that the synchronization of stimulation boosts the system to reach the threshold. The characteristics of prosodic information, namely synchronization with syntactic boundaries and the automatic capture of attention, might thus contain the relevant requisites to facilitate the extraction and generalization of the rules embedded in speech. This rhythmic cue might also facilitate the synchronization of neural activity (Arnal and Giraud, 2012), given that learners tuned to extract words synchronize at different frequency bands than those tuned to learn rules from the same language (de Diego-Balaguer et al., 2011).

In summary, the presence of pauses provoked two functionally distinct dissociations: an N1 attenuation immediately triggered by the presence of the pause cue enabling segmentation and a P2 modulation that was not automatically triggered by pauses but was associated to learning and was enhanced when this cue could be used to learn the embedded rules. In their seminal study, Peña et al. (2002) proposed that the presence of pauses changes the computations applied to the speech signal, facilitating the extraction and generalization of the embedded rules: “*A system looking for structure in speech is naturally attuned to a signal modulated by rhythm and intonation.”* Humans are tuned to speech and are sensitive to the prosodic characteristics of language at an early stage. This bias might exploit the modulation of endogenous and exogenous mechanisms to facilitate the segmentation of speech and the extraction of syntactic rules from language.

## Conflict of Interest Statement

The authors declare that the research was conducted in the absence of any commercial or financial relationships that could be construed as a potential conflict of interest.

## Footnotes

1 http://tcts.fpms.ac.be/synthesis/mbrola.html

2 http://nbs.neurobs.com/
